# Characterization of two reductases *MaLAR* and *MaANR* revealed their roles in proanthocyanidin biosynthesis in mulberry

**DOI:** 10.3389/fpls.2025.1760417

**Published:** 2026-01-21

**Authors:** Zhiheng Feng, Peijun Li, Guang Yang, Yining Wang, Mengqi Li, Jiangting Wu, Nan Chao, Li Liu

**Affiliations:** 1 Jiangsu Key Laboratory of Sericultural Biology and Biotechnology, School of Biotechnology, Jiangsu University of Science and Technology, Zhenjiang, Jiangsu, China; 2 Key Laboratory of Silkworm and Mulberry Genetic Improvement, Ministry of Agriculture and Rural Affairs, Sericulture Research Center, Chinese Academy of Agricultural Sciences. Zhenjiang, Jiangsu, China

**Keywords:** anthocyanidin reductase (ANR), biosynthesis, fruit development, functional characterization, leucoanthocyanidin reductase (LAR), mulberry (Morus), proanthocyanidins (PAs)

## Abstract

Proanthocyanidins (PAs), polymers of flavan-3-ols, are crucial for the sensory quality and stress defense of mulberry (*Morus* spp.). The biosynthesis of their monomers, catechin and epicatechin, are catalyzed by leucoanthocyanidin reductase (*LAR*) and anthocyanidin reductase (*ANR*), respectively, representing key rate-limiting steps that determine PA composition and abundance. In this study, systematic functional analyses including phylogenetic analysis, quantified spatio-temporal expression profiles during fruit development (S1-S4 stages), *in vitro* enzymatic assay, knock-down using Virus-Induced Gene Silencing (VIGS) in mulberry leaves and heterologous overexpression in *Arabidopsis thaliana* were conducted to reveal their roles in proanthocyanidin biosynthesis in mulberry. Results showed that *MaLAR* (969 bp) and *MaANR* (1014 bp) were successfully cloned and phylogenetically conserved. Spatio-temporal expression analysis revealed distinct patterns: *MaLAR* expression continuously increased, reaching highest expression level at the fully ripe stage (S4), whereas *MaANR* showed highest expression level at the color-turning stage (S2). *In vitro* enzymatic assays confirmed that *MaLAR* catalyzed the formation of catechin from leucoanthocyanidin, and *MaANR* catalyzed the formation of epicatechin from anthocyanidin. VIGS-mediated silencing of either gene in mulberry leaves led to significant reduction in total PA content. Conversely, heterologous overexpression of *MaLAR* or *MaANR* in *Arabidopsis* resulted in significant increase of PA in the seed coat. Our findings confirm that *MaLAR* and *MaANR* are conserved, key positive regulators of PA biosynthesis in mulberry. The differential expression patterns during fruit ripening suggest they play distinct, temporally regulated roles in determining the final PA composition and content. These results provide an important theoretical basis and represent important targets for the metabolic engineering and molecular breeding of mulberry for improved fruit quality.

## 1 Introduction

Proanthocyanidins (PAs), also known as condensed tannins, are a class of secondary metabolites widely present in plants (Marles et al., 2003). They are polymers formed through the C-C bond condensation of flavan-3-ol monomers, primarily catechin and epicatechin (Rauf et al., 2019). In plants, PAs play vital roles in defense mechanisms and stress adaptation, offering protection against UV radiation, pathogens, and herbivores (He et al., 2008). Furthermore, PAs are critical determinants of the sensory quality of fruits, vegetables, and beverages, significantly influencing color stability, astringency, and mouthfeel (Yu et al., 2020). For human health, PAs are valued for their potent antioxidant properties and potential benefits in preventing chronic diseases (Xie et al., 2003).

Mulberry (*Morus alba* L.), a woody plant cultivated globally for its economic, edible, and medicinal value, accumulates a significant number of PAs in its fruits and leaves (Xin et al., 2020). The profile and concentration of these compounds are directly linked to the fruit’s sensory quality, processing suitability, and potential health benefits. Moreover, PA accumulation is hypothesized to contribute to phenotypic variations and stress resilience among different mulberry varieties (Jin et al., 2017).

The biosynthesis of PAs is a distinct branch of the flavonoid metabolic pathway, sharing precursors with anthocyanin synthesis. Two key rate-limiting enzymes, leucoanthocyanidin reductase (LAR, EC 1.17.1.3) and anthocyanidin reductase (ANR, EC 1.3.1.112), are crucial in determining the monomeric composition and abundance of PAs (Lu, 2024). LAR catalyzes the reduction of leucoanthocyanidins (e.g., leucocyanidin) to form 2,3-trans-flavan-3-ols (catechin), while ANR converts anthocyanidins (e.g., cyanidin) into 2,3-cis-flavan-3-ols (epicatechin) (He et al., 2008). The coordinated action of LAR and ANR thus governs the catechin/epicatechin ratio, which in turn dictates the degree of polymerization, structural isomerism, and the terminal/extension unit composition of the final PA polymer. These physicochemical characteristics ultimately define the fruit’s astringency, color stability, and processing attributes (Rauf et al., 2019; Zhao et al., 2023; Kennedy et al., 2001).

While the functions of LAR and ANR have been extensively studied in model plants like *Arabidopsis* (Marinova et al., 2007) and commercially important crops such as grape (*Vitis vinifera*) (Bogs et al., 2007), Persimmon (*Diospyros kaki*), and Litchi (*Litchi chinensis* Sonn.) (Zhong et al., 2024), systematic identification and functional validation of these enzymes in mulberry remain notably insufficient. Validated functional evidence still needs to be provided for identifying and characterizing LAR and ANR to reveal their roles in proanthocyanidin biosynthesis.

In the present study, we identified and cloned the candidate genes *MaLAR* and *MaANR* from *M. atropurpurea* var. *‘Zhongshen No.1’* (Mazs), and then conducted detailed sequence conservation and phylogenetic analyses. Spatiotemporal expression patterns in various tissues and during fruit ripening were characterized. enzymatic assay indicated *MaLAR* and *MaANR* were responsible for catechin and epicatechin formation respectively in mulberry. Further transgenic analyses revealed that they are positive regulators for PA biosynthesis.

## 2 Materials and methods

### 2.1 Plant materials and sample collection

The mulberry variety used in this study was *M. atropurpurea* var. *‘Zhongshen No.1’* (Mazs). The plants were cultivated and maintained at the National Mulberry Germplasm Resource Nursery (Zhenjiang, China; 32°12′ N, 119°27′ E), affiliated with the Sericultural Research Institute of the Chinese Academy of Agricultural Sciences (CAAS) and Jiangsu University of Science and Technology (JUST).

Tender leaves collected from the shoot apices were immediately frozen in liquid nitrogen and stored at -80 °C for subsequent total RNA extraction and gene cloning.

Mulberry fruit samples (cv. Mazs) were collected at four distinct developmental stages based on ripening time and phenotype: the green-ripe stage (S1, 7 days post-anthesis, DPA), the color-turning stage (S2, 14 DPA), the mature stage (S3, 21 DPA), and the fully-ripe stage (S4, 28 DPA) (Fu et al., 2025). All harvested fruit samples were immediately snap-frozen in liquid nitrogen and stored at -80°C until use.

### 2.2 Methods

#### 2.2.1 Gene cloning and bioinformatics analysis

Amino acid sequences of *LAR* and *ANR* from *Arabidopsis thaliana*, *Vitis vinifera* L., and *Camellia sinensis* L. were used as queries for a BLASTP search against the *M. notabilis* genome (https://*Morus*.biodb.org/*Morus*db) (He et al., 2013) (E-value ≤ 1 × 10^−20^, top 10% score). This search identified the candidate genes L484_022305.p01 (designated *MnLAR)* and L484_014834.p01 (designated *MnANR*).

Specific primers were designed based on the coding sequences (CDS) of the target genes, flanked with sequences compatible with seamless cloning using the pNC-Blunt vector system. Total RNA was extracted from tender leaves of the ‘Mazs’ cultivar using the EASYspin Plus Complex Plant RNA Kit (RN5301, Aidlab, Beijing, China). First-strand cDNA was synthesized with the HiScript II Q RT SuperMix for qPCR (+gDNA wiper) kit (R223-01, Vazyme, Nanjing, China), following the manufacturer’s instructions. The full-length CDS of *MaLAR* (969 bp) and *MaANR* (1014 bp) were amplified using a high-fidelity DNA polymerase. The PCR products (*MaLAR* and *MaANR* CDS; see **Supplementary Figure S1**) were confirmed by 1% agarose gel electrophoresis, purified using the SanPrep Column DNA Gel Extraction Kit (B518131, Sangon, Shanghai, China), and subsequently ligated into the pNC-Blunt vector using the Ready-to-Use Seamless Cloning Kit (GS2288, Bai’ao Laibo, Beijing, China). The resulting constructs were transformed into *Escherichia coli* TOP10 competent cells. Positive clones were selected and verified by Sanger sequencing, yielding the final constructs *MaLAR* and *MaANR* derived from the ‘Mazs’ variety.

#### 2.2.2 Spatiotemporal expression analysis by qRT-PCR

Total RNA was extracted from tender leaves and fruit at four developmental stages (S1, S2, S3, S4) of ‘Mazs’, with three biological replicates per tissue type. RNA quality and concentration were assessed using a NanoDrop spectrophotometer (A260/A280 and A260/A230 ratios), and integrity was verified by 1% agarose gel electrophoresis. Genomic DNA was removed using gDNA Eraser, and first-strand cDNA was synthesized using the HiScript III 1st Strand cDNA Synthesis Kit (+gDNA wiper) (R312-01, Vazyme, Nanjing, China).

qPCR primers were designed using the Primer-BLAST platform (amplification efficiency 95–105%, the primer sequences for *MaLAR* and *MaANR* are provided in **Supplementary Table S1**). Mulberry *Actin* (Shukla et al., 2019) GenBank: HQ123583) was used as the internal reference gene. qRT-PCR reactions were performed using SYBR^®^ Premix Ex Taq™ II (Q341-02, Vazyme, Nanjing, China) in a 20 μL system (10 μL Premix, 0.4 μM of each primer, 2 μL cDNA) on an ABI 7500 Real-Time PCR System. The thermal cycling conditions were: 95°C for 30 s; 40 cycles of 95°C for 5 s and 60°C for 34 s (fluorescence acquisition). A melt curve analysis (95°C for 15 s, 60°C for 1 min, 95°C for 15 s) was conducted to ensure amplification specificity. Three technical replicates were performed for each sample. Relative expression levels were calculated using the 2^−ΔΔCt^ method and are presented as the mean ± SD.

#### 2.2.3 Recombinant protein expression, purification, and HPLC-based enzyme assay

The sequence-verified CDS of *MaLAR* and *MaANR* were cloned into the pET-28a (+) vector (N-terminal His_6_ tag) and transformed into E. coli BL21 (DE3) cells. Cultures were grown at 37°C in LB medium (50 μg/mL kanamycin) to an OD_600_ of 0.6–0.8. Protein expression was induced with 0.5 mM IPTG and incubated overnight at 16°C.

Cells were harvested, resuspended in lysis buffer, and disrupted by sonication. The soluble fraction was purified by Ni^2+^-NTA affinity chromatography, followed by dialysis to remove free imidazole. Protein purity and molecular weight were confirmed by SDS–PAGE (**Supplementary Figure S2**).

The *in vitro* enzymatic assays for MaLAR and MaANR were performed as previously described, with minor modifications (Gagné et al., 2009). For the MaLAR assay, taxifolin (dihydroquercetin; V91397, MedMol, Shanghai, China) was used as the substrate. The reaction mixture (300 µL total volume) contained 5 µL taxifolin (2000 ng/µL), 152 µL 0.1 M Tris-HCl buffer (pH 7.5; T301503, Aladdin, Shanghai, China), 2 µL NADPH (N276326, Aladdin, Shanghai, China), 70 µL purified MaDFR1, 70 µL purified MaLAR, and 1 µL dithiothreitol (DTT; D755747, Aladdin, Shanghai, China). For the MaANR assay, cyanidin chloride (B20784, MedMol, Shanghai, China) was used as the substrate. The reaction mixture (500 µL total volume) contained 100 µL cyanidin chloride (2000 ng/µL), 195 µL 0.1 M Tris-HCl buffer (pH 6.0; T301503, Aladdin, Shanghai, China), 5 µL NADPH (N276326, Aladdin, Shanghai, China), and 200 µL purified MaANR. Control reactions were performed using heat-inactivated enzymes. Reactions were incubated in a constant-temperature water bath at 35 °C for 45 min. Reaction products were extracted with ethyl acetate (E116141, Aladdin, Shanghai, China) by vortexing (the extraction step for ANR assays was repeated once). The combined organic phases were dried under nitrogen, redissolved in HPLC-grade methanol (M433267, Aladdin, Shanghai, China), centrifuged, and filtered prior to HPLC analysis.

Enzymatic products were detected at 290 nm. Substrate consumption and product formation were quantified by comparing retention times with authentic standards using the external standard method.

#### 2.2.4 Virus-Induced Gene Silencing and knock-down analysis

Gene-specific 300 bp fragments of *MaLAR* and *MaANR* were predicted using the online SGN VIGS Tool (https://vigs.solgenomics.net/), and the primers used for gene silencing are listed in **Supplementary Table S1**. Fragments were amplified with primers containing NC-linker arms, verified by electrophoresis, and ligated into the linearized pNC-TRV2-GFP vector via seamless cloning to construct pNC-TRV2-GFP-*MaLAR* and pNC-TRV2-GFP-*MaANR*.

The recombinant TRV2 plasmids and the TRV1 vector were co-transformed (1:1 molar ratio) into *Agrobacterium tumefaciens* GV3101. Cultures were grown to OD_600_ ≈ 1.0, collected, and resuspended in MMA infiltration buffer (10 mM MgCl_2_, 10 mM MES, pH 5.6, 150 μM acetosyringone), and incubated at room temperature for 3 h (Mo et al., 2024).

Sterile seedlings of *M alba* var. *fengchi* (4–6 true leaves) were infiltrated on the abaxial leaf side via syringe. Plants were grown at 24°C (16 h light/8 h dark). GFP fluorescence was monitored 2–3-week post-infiltration under UV light to select positive plants. Total RNA was extracted from GFP-positive leaves, and transcript levels of *MaLAR* and *MaANR* were quantified by qRT-PCR (Actin internal control) using the 2^−ΔΔCt^ method to evaluate gene silencing efficiency. (**Supplementary Figure S3**).

#### 2.2.5 Generation of overexpression *Arabidopsis* and phenotypic analysis

The full-length CDSs of *MaLAR* (969bp) and *MaANR* (1,014bp) were cloned into the pNC-Cam13FN vector under the control of the CaMV 35S promoter. Recombinant plasmids were introduced into *A. tumefaciens* GV3101 via triparental mating. *A. thaliana* (Col-0) plants were transformed using the floral dip method (Clough and Bent, 1998).

Harvested T_0_ seeds were screened on 1/2 MS medium containing 50 μg/mL kanamycin. Positive transformants were grown and self-pollinated to obtain T_2_ generation seeds.

Transgene integration in T_2_ plants was confirmed by genomic DNA PCR using gene-specific primers identical to those employed for qRT-PCR analysis. Transgene expression levels were quantified by qRT-PCR using cDNA as the template, with *AtACT7* as the internal control. Relative expression was calculated using the 2^−ΔΔCt^ method. All primer sequences used for genomic DNA PCR and qRT-PCR analyses are listed in **Supplementary Table S1**. PA distribution in T_2_ seed coats was visualized by staining seeds with 0.1% DMACA solution for 10 min and observing them under an optical microscope. Total PA content in seedlings was quantified colorimetrically using the DMACA method (640 nm absorbance) (Li et al., 1996), with epicatechin as the standard.

## 3 Results

### 3.1 Identification and characterization of mulberry LAR and ANR

Using the protein sequences of *LAR* and *ANR* from *A. thaliana*, *V. vinifera*, and *C. sinensis* as queries, BLASTP searches were performed against the *M. notabilis* genome. Two candidate genes were identified: L484_022305.p01 and L484_014834.p01, which were subsequently designated as *MnLAR* and *MnANR*, respectively (**Table 1**). *MaLAR* and *MaANR* were then successfully cloned from *M. atropurpurea* var. *Zhongshen No.1* (Mazs). The CDS of *MaLAR* was 969 bp in length, encoding a protein of 322 amino acids, whereas the CDS of *MaANR* was 1,014 bp, encoding a 337-amino-acid protein. The sequences of MaANR and MaLAR analyzed in this study have been deposited in the GenBase database (NGDC; https://ngdc.cncb.ac.cn/genbase/) under the accession numbers C_AA132743.1 and C_AA132744.1, respectively.

**Table 1:** Screening Results of *M. notabilis*.

NO.	Gene ID	E-value	Score	Gene name
1	L484_022305.p01	3e-80	658	*MnLAR*
2	L484_014834.p01	6e-90	679	*MnANR*

**Figure 1:**
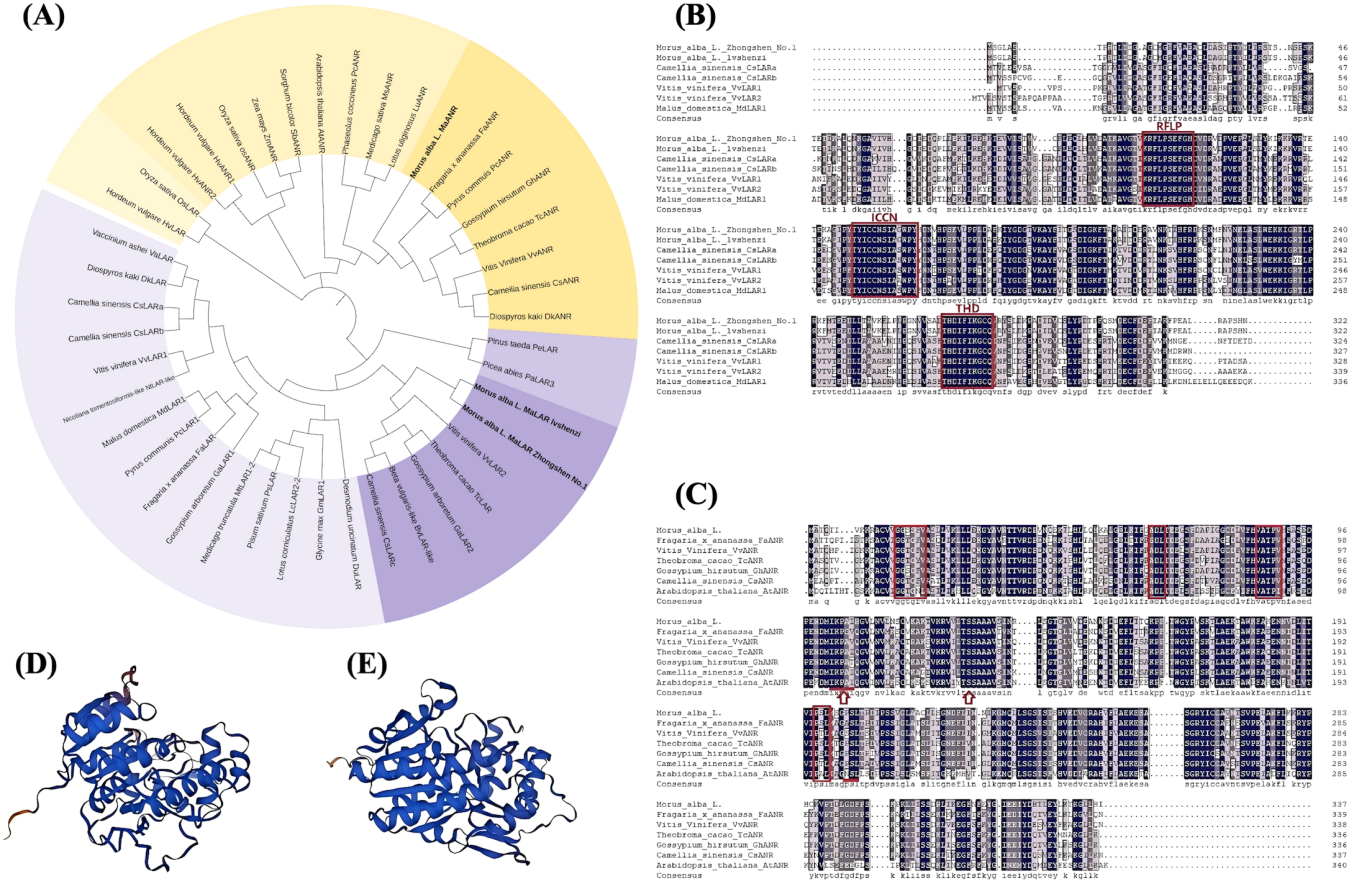
Cloning and identification of *MaLAR* and *MaANR* genes in mulberry. **(A)** Phylogenetic tree of MaLAR and MaANR proteins from mulberry aligned with homologous amino acid sequences from other species. The MaLAR and MaANR are indicated in bold. **(B)** Multiple sequence alignment of LAR from mulberry and other species. The three conserved domains are indicated by square boxes. **(C)** Multiple sequence alignment of MaANR from mulberry and other species. The arrow, square, and horizontal line indicate the predicted active site, the NADPH coenzyme-binding site, and the substrate-binding site, respectively. **(D)** Predicted tertiary structure of the MaLAR protein from mulberry. **(E)** Predicted tertiary structure of the MaANR protein from mulberry.

Phylogenetic analysis showed that MaANR and MaLAR clustered with previously characterized ANR and LAR proteins from other plant species, respectively (**Figure 1A**). Multiple sequence alignment and conserved domain analysis revealed that *MaANR* contains the conserved NADPH-binding site, and its predicted substrate-binding residues are highly consistent with those of reported ANRs. *MaLAR* harbors the three conserved motifs RFLP, ICCN, and THD, which are characteristic of this reductase family (Wang et al., 2018; Liao et al., 2015; Ferraro et al., 2014) (**Figures 1B, C**). Homology-based 3D structural modeling indicated clear structural differences between *MaANR* and *MaLAR*, consistent with their distinct catalytic functions (**Figure 1D**).

### 3.2 Spatiotemporal expression patterns of *MaLAR* and *MaANR*

**Figure 2:**
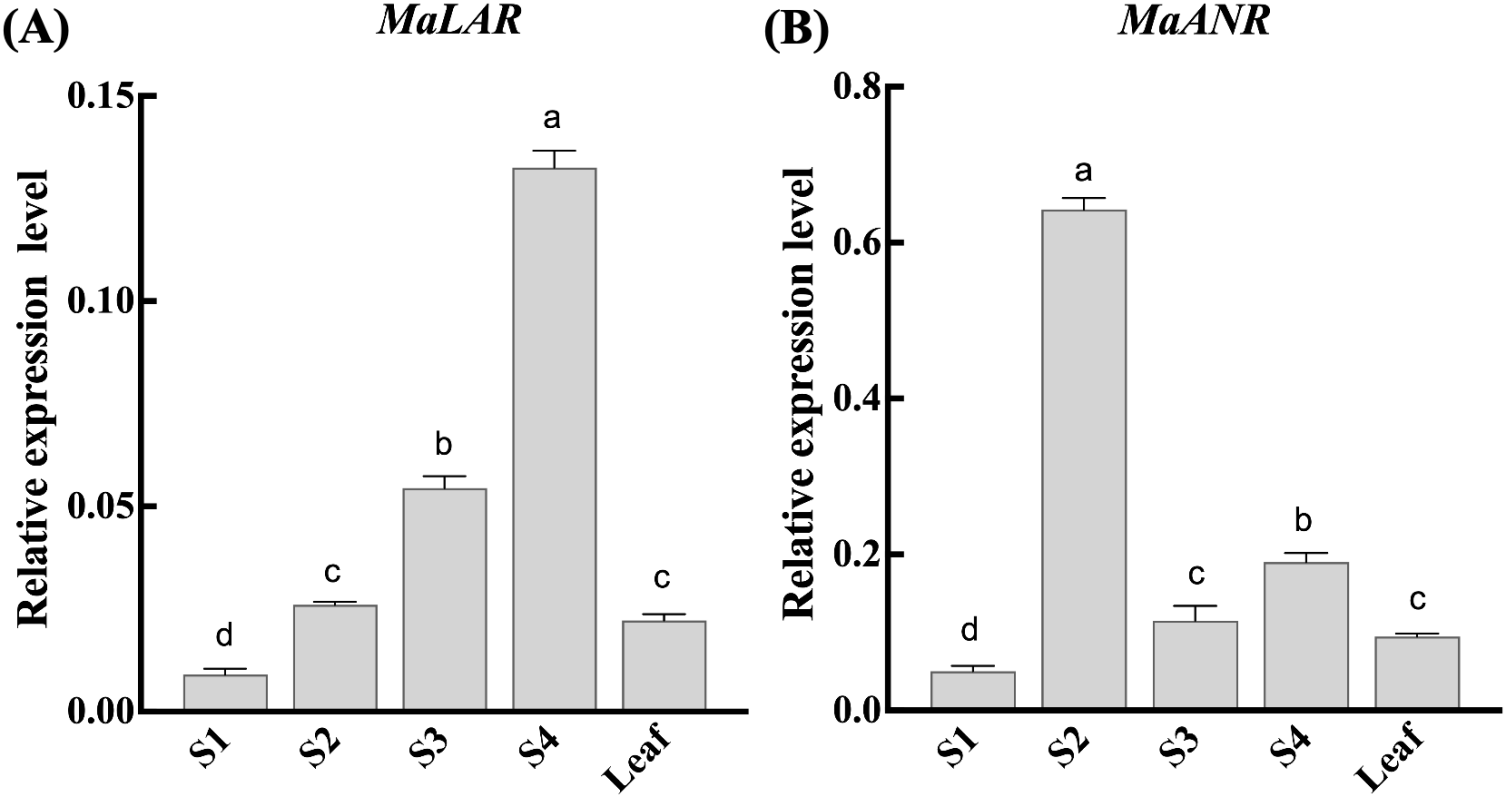
Analysis of *MaLAR* and *MaANR* gene expression levels at different developmental stages. **(A)***MaLAR* organ expression profile. **(B)***MaANR* organ expression profile. S1 Green-ripe stage; S2 Color-turning stage; S3 Mature stage; S4 Fully-ripe stage; Leaf. Values represent the mean ± SD of three biological replicates (n = 3). Different lowercase letters above the bars indicate statistically significant differences among samples, as determined by one-way ANOVA followed by Tukey’s multiple comparison test (*P* < 0.05).

The spatiotemporal expression patterns of *MaLAR* and *MaANR* were assessed by quantitative real-time PCR (qRT-PCR) using cDNA synthesized from Ma leaves and fruits collected at four developmental stages (S1, S2, S3, S4) (**Figure 2**). Both genes displayed pronounced tissue specificity and dynamic transcriptional changes during fruit maturation.

The expression of *MaLAR* increased progressively throughout fruit development, reaching its maximum at the fully ripe stage (S4) (**Figure 2A**). In contrast, *MaANR* exhibited transient induction pattern, with transcript levels peaking at the color-turning stage (S2) before declining at later stages (**Figure 2B**).

These distinct expression trajectories indicate that *MaLAR* and *MaANR* may fulfill complementary yet non-redundant functions in regulating proanthocyanidin biosynthesis during mulberry fruit development.

### 3.3 *In vitro* enzymatic assay of *MaLAR* and *MaANR*

**Figure 3:**
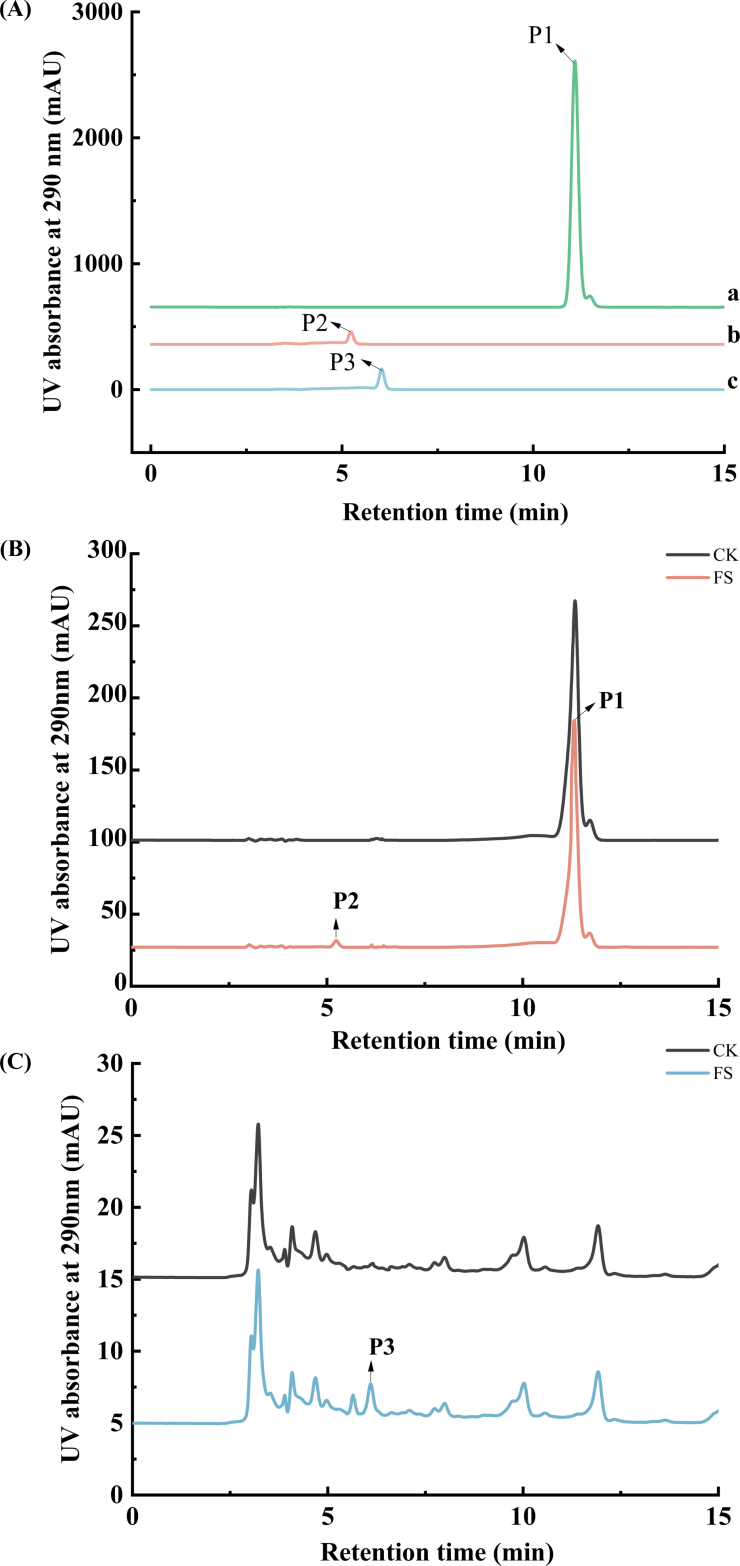
Chromatogram of *MaLAR* and *MaANR* enzymatic assay. **(A)** Chromatograms of dihydroquercetin, catechin and epicatechin standards; A-a, dihydroquercetin; A-b, Catechins; A-c, epicatechin. **(B)** Chromatogram of *MaLAR* enzyme catalytic activity assay. FS: Experimental group, Reaction system + *MaLAR*; CK: Control group, Reaction system + *MaLAR* (heat-inactivated). **(C)** Chromatogram of *MaANR* enzyme catalytic activity assay. FS: Reaction system + *MaANR*; CK: Reaction system + *MaANR* (heat-inactivated).

To further validate the roles of *MaLAR* and *MaANR* in the PA biosynthetic pathway, recombinant proteins were expressed and purified, and *in vitro* enzymatic assay systems were established. The purity and molecular weights of the purified proteins were confirmed by SDS–PAGE (**Supplementary Figure S2**), and these proteins were then used for enzymatic activity assays. The standard products, catechin and epicatechin serving as references for product identification were detected using HPLC-VWD at 290 nm with retention time of 5.226 min and 6.035 min respectively, (**Figure 3A**). Enzymatic assay showed that MaLAR can catalyze leucoanthocyanidin produced by DFR using dihydroquercetin as substrates (**Figure 3B**). Furthermore, the MaANR activity assay revealed a characteristic epicatechin peak at 6.072 min. This product was also not detected in the heat-inactivated control (**Figure 3C**), confirming its ability to reduce anthocyanidin to epicatechin.

Collectively, the *in vitro* enzymatic evidence unequivocally confirms that *MaLAR* and *MaANR* are responsible for the formation of catechin and epicatechin, respectively, further establishing their critical and rate-limiting roles in the mulberry PA biosynthetic pathway (Sunkar et al., 2003; Tucker, 2003; Pang et al., 2013).

### 3.4 Knock-down of either *MaLAR* or *MaANR* using VIGS resulted in reduction of PAs

To elucidate the *in vivo* functional roles of *MaLAR* and *MaANR* in the mulberry PA biosynthetic pathway, virus-induced gene silencing (VIGS) constructs (pNC-TRV2-GFP-*MaLAR* and pNC-TRV2-GFP-*MaANR*) were generated and introduced into *M. alba* var. *fengchi*.

**Figure 4:**
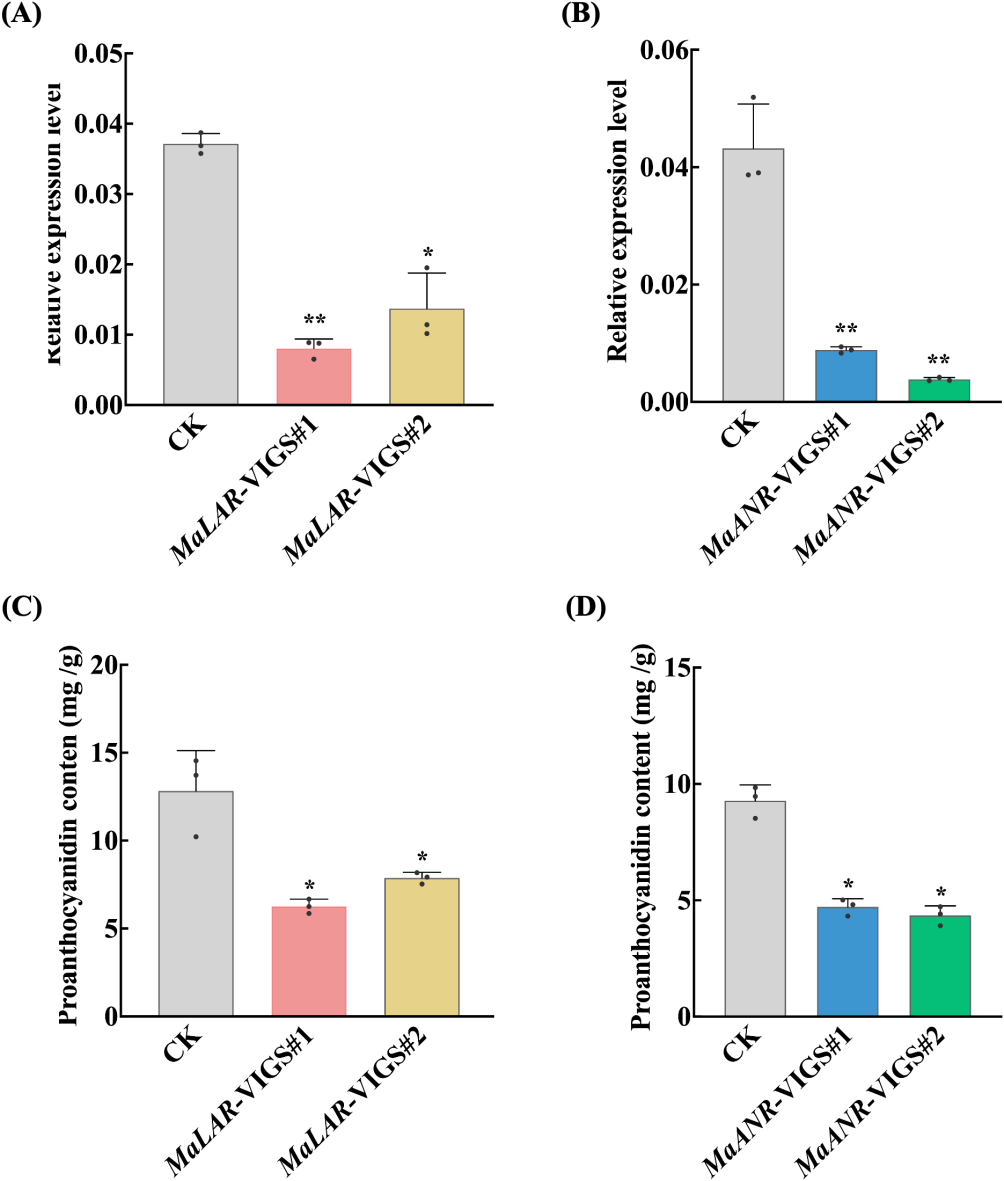
Changes in gene expression levels and proanthocyanidin content after VIGS treatment. **(A)** Expression levels of *MaLAR* in mulberry leaves after VIGS treatment. **(B)** Expression levels of *MaANR* in mulberry leaves after VIGS treatment. **(C)** Proanthocyanidin content in leaves of *MaLAR*-knockdown lines. **(D)** Proanthocyanidin content in leaves of *MaANR*- knockdown lines; Statistical significance was assessed using Student’s *t*-test. Significance levels were indicated as follows: 0.01 < *P* ≤ 0.05 (*), and 0.001 < *P* ≤ 0.01 (**).

qRT-PCR analysis verified effective suppression of the target genes in the silenced lines. *MaLAR* transcript levels were reduced by 78.28% and 63.06% in lines #1 and #2, respectively (**Figure 4A**). Similarly, *MaANR* expression decreased by 79.48% and 91.11% in the corresponding #1 and #2 lines (**Figure 4B**).

Consistent with the extent of gene silencing, total PA levels also showed pronounced declines. Relative to the control plants, silencing *MaLAR* reduced total PAs content by 51.16% and 38.57% in lines #1 and #2, respectively (**Figure 4C**). Silencing *MaANR* resulted in decreases of 49.15% and 53.11% in lines #1 and #2, respectively (**Figure 4D**). Given that catechin and epicatechin serve as essential monomeric precursors for PA polymerization, impaired synthesis of these intermediates directly contributed to the diminished PA accumulation observed in the silenced plants.

Collectively, these VIGS assays provide strong evidence for *MaLAR* and *MaANR* as key *in vivo* contributors to PA biosynthesis in mulberry, and downregulation of either gene leads to substantial reduction in total PA content.

### 3.5 Heterologous overexpression of *MaLAR* and *MaANR* in *A. thaliana* resulted in accumulation of PAs

To further substantiate the functional roles of *MaLAR* and *MaANR* in PA biosynthesis, each genes was heterologously overexpressed in *A. thaliana* independently. The resulting transgenic lines were systematically evaluated for phenotypic characteristics, transcriptional changes, and metabolite accumulation.

**Figure 5:**
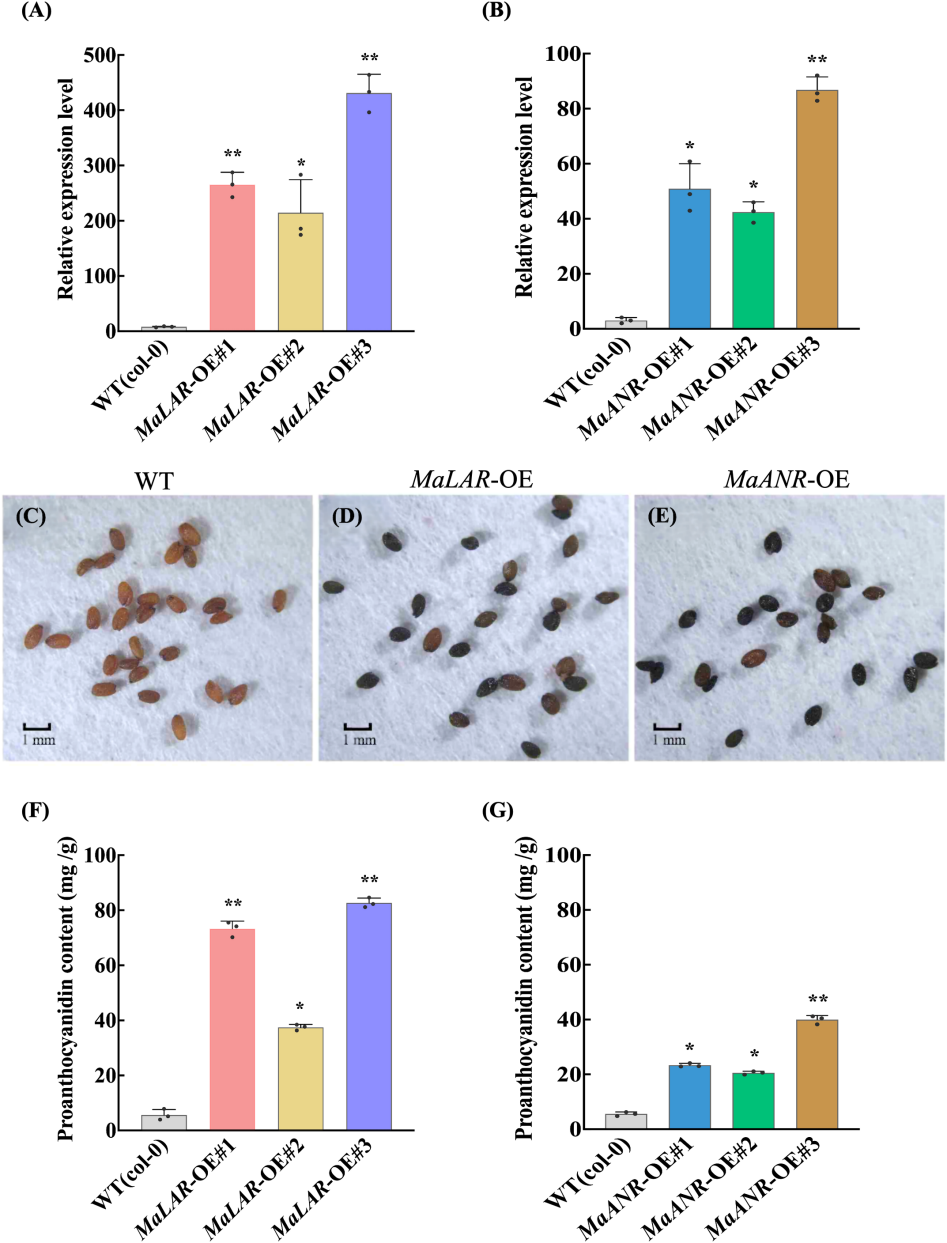
OE-*Arabidopsis* seed staining and PAs quantification. **(A)** Expression level of *MaLAR* in transgenic *Arabidopsis***(B)** Expression level of *MaANR* in transgenic *Arabidopsis***(C)** Wild-type *Arabidopsis thaliana* seeds **(D)** Overexpression of *MaLAR A. thaliana* seeds **(E)** Overexpression of *MaANR A. thaliana* seeds **(F)** Proanthocyanidin content determination in *MaLAR*-overexpressing *Arabidopsis***(G)** Proanthocyanidin content in *MaANR*-overexpressing *Arabidopsis.* Statistical significance was assessed using Student’s *t*-test. Significance levels were indicated as follows: 0.01 < *P* ≤ 0.05 (*), and 0.001 < *P* ≤ 0.01 (**).

qRT-PCR analysis confirmed substantial upregulation of the target genes, with *MaLAR* transcript levels elevated by 25.54- to 52.37-fold and *MaANR* levels increased by 12.93- to 27.53-fold relative to the wild type (WT) (**Figures 5A, B**). DMACA staining of T_2_ seeds revealed markedly intensified blue coloration in the transgenic lines compared with WT seeds (**Figures 5C–E**), indicating enhanced PA deposition in the seed coat.

Consistent with the staining results, quantitative measurements showed dramatic increases in total PA content. In the *MaLAR* overexpression lines, PA levels were 5.61- to 13.58-fold higher than in WT, whereas *MaANR* overexpression lines exhibited 2.69- to 6.18-fold increases (**Figures 5F, G**). This substantial accumulation is attributable to the heightened synthesis of catechin and epicatechin—key monomeric precursors required for PA polymerization—thereby driving elevated PA deposition and intensified staining signals.

In conclusion, the heterologous overexpression results in *Arabidopsis* demonstrate that increased transcription of *MaLAR* and *MaANR* markedly enhances the biosynthetic flux toward catechin and epicatechin, ultimately promoting PA accumulation in the seed coat. These findings, in concordance with the VIGS silencing assays and the *in vitro* enzymatic characterization, collectively confirm the essential positive regulatory roles of *MaLAR* and *MaANR* in the mulberry PA biosynthetic pathway.

## 4 Discussion

### 4.1 Core functions of *LAR* and *ANR* in proanthocyanidin biosynthesis

LAR and ANR are key terminal enzymes in the PA biosynthetic pathway, catalyzing the formation of catechin and epicatechin, respectively (Jun et al., 2021). By controlling both the availability and stereochemical configuration of flavan-3-ol monomers, these enzymes play central roles in shaping PA composition and structural diversity. Although the core PA biosynthetic pathway is broadly conserved among higher plants, increasing evidence has revealed substantial enzymatic complexity and functional diversification of LAR and ANR across species (Yu et al., 2023; Lin et al., 2024).

Notably, in several plant species, ANR exhibits expanded catalytic activities, generating not only epicatechin starter units but also putative intermediates such as 2,3-*trans*-flavan-3,4-diols or their corresponding thiol derivatives, which can serve as extension units during PA polymerization (Jun et al., 2021). Such substrate versatility highlights the species-specific catalytic preferences of ANR and underscores its influence on PA polymer length and composition. Similarly, in species such as grapevine and *Medicago truncatula*, LAR has been reported to catalyze not only the conversion of leucoanthocyanidins to catechin but also the reverse transformation of 4β-(*S*-cysteinyl)-epicatechin to epicatechin. This capacity modulates the balance between starter and extension units, ultimately shaping PA chain length (Xie et al., 2004; Liang et al., 2023).

These functional characteristics contrast sharply with the metabolic landscape of *Arabidopsis thaliana*, which lacks an endogenous *LAR* gene and accumulates proanthocyanidins composed almost exclusively of epicatechin units derived from the ANR pathway (Gagné et al., 2009; Chen et al., 2022; Bogs et al., 2005). In the present study, however, heterologous expression of *MaLAR* or *MaANR* in *Arabidopsis* resulted in a significant increase in total PA content, as quantified by the DMACA colorimetric assay. Although this method does not discriminate between catechin- and epicatechin-derived units, it reliably reflects overall PA accumulation. The observed increase in PA levels suggests that the introduction of functional MaLAR activity—which is otherwise absent in *Arabidopsis*—may enhance the overall flux of flavan-3-ols toward PA biosynthesis. Crucially, such enhancement does not necessarily require detectable accumulation of free catechin monomers, as newly formed catechin units may be rapidly incorporated into PA polymers or indirectly influence PA production by redirecting metabolic intermediates away from competing flavonoid branches.

Previous research has reported that co-expression of multiple flavonoid biosynthetic genes can enable ectopic catechin biosynthesis, as demonstrated by the simultaneous expression of *CsANS*, *CsLAR*, and *CsANR* from the tea plant in tobacco (Yang et al., 2025). In addition, the *ban* and *ldox* mutants of *Arabidopsis thaliana* are well-characterized lines impaired in proanthocyanidin biosynthesis due to defects in key enzymatic steps of the flavonoid pathway, making them suitable genetic backgrounds for complementation-based functional validation (Abrahams et al., 2002; Rafique et al., 2016). Accordingly, genetic complementation using *Arabidopsis ban* or *ldox* mutants, together with co-expression of *MaLAR* and *MaANR*, could be explored in future work to further substantiate their roles in PA biosynthesis. Consistent with this interpretation, previous studies in species possessing functional LAR, including cacao and grapevine, have demonstrated that LAR activity contributes to PA accumulation primarily through modulation of flavonoid metabolic flux rather than through substantial accumulation of free catechin monomers. Collectively, these findings support the notion that, although the catalytic functions of LAR and ANR are central to PA biosynthesis across diverse plant lineages, species-specific metabolic contexts ultimately determine PA composition and structural complexity (Chen et al., 2022; Bogs et al., 2005; He et al., 2008; Lin et al., 2024; Liu et al., 2013; Yu et al., 2023).

### 4.2 Evolutionary patterns and regulatory networks of *LAR* and *ANR* genes

At the genomic level, *LAR* and *ANR* genes are broadly distributed across angiosperms; however, their copy number and structural organization frequently exhibit species-specific expansion and diversification (Jiao et al., 2023). In several economically important crops, such as grape and tea, multiple functional homologs of *LAR* and *ANR* have been identified, and their expression levels show strong correlations with the accumulation of catechin and epicatechin (Lin et al., 2024).

The spatiotemporal transcriptional regulation of *LAR* and *ANR* is primarily orchestrated by the MYB–bHLH–WD40 (MBW) regulatory complex (Shan et al., 2025). In the *Arabidopsis* seed coat, the TT2–TT8–TTG1 module constitutes the core regulatory unit that activates *ANR* expression, and homologous regulatory components have been identified and shown to be functionally conserved in species such as grape, strawberry, and tobacco (Jiang et al., 2023).

Beyond the canonical MBW complex, hormonal cues (e.g., auxin and ethylene) and environmental stimuli (e.g., UV-C irradiation) can further modulate *LAR* and *ANR* expression (Bulanov et al., 2025). For example, in apple, ethylene-responsive transcription factors indirectly enhance Md*LAR* and Md*ANR* expression through upstream regulation of MYB activators (Wang et al., 2022b).

Collectively, these findings indicate that *LAR* and *ANR* gene regulation operates through multilayered and species-specific networks, reflecting divergent evolutionary strategies that enable plants to meet distinct biochemical and physiological demands for PA biosynthesis under developmental and environmental constraints (Jiang et al., 2023).

### 4.3 Application potential and future directions of PA metabolic engineering

With the growing recognition of the roles of PAs in enhancing plant stress resistance and nutritional quality (Yu et al., 2023), metabolic engineering targeting *LAR* and *ANR* has emerged as an effective strategy for crop trait improvement (Jiang et al., 2023). The heterologous expression of MYB, bHLH, and LAR/ANR genes has enabled the establishment of PA-accumulating systems in plants that typically do not synthesize PAs (Escaray et al., 2023). For instance, robust PA production has been achieved in tobacco leaves and white clover, resulting in enhanced nitrogen use efficiency and reduced methane emissions in ruminant livestock (Lu et al., 2023). In addition, upregulation of LAR/ANR in crops such as cotton and mulberry has been shown to improve disease resistance and promote fiber development (Wang et al., 2022a).

Despite these advances, excessive PA accumulation can negatively affect plant growth. Therefore, recent studies have increasingly focused on tissue-specific or inducible expression systems to mitigate potential developmental penalties associated with constitutive overaccumulation (Khan et al., 2023). Looking forward, comprehensive elucidation of the *LAR/ANR* regulatory network, together with emerging technologies such as gene editing and precision transcriptional regulation, will further facilitate the development of optimized PA metabolic engineering strategies aimed at improving crop quality, enhancing stress resilience, and increasing the nutritional value of forage crops (Das et al., 2024; Wang et al., 2025).

## 5 Conclusion

This study leveraged *M. notabilis* genomic resources to comprehensively elucidate the molecular characteristics and functional roles of the key reductases *MaLAR* and *MaANR* in the mulberry proanthocyanidin (PA) biosynthetic pathway.

Our results demonstrate that both genes are highly conserved in mulberry and exhibit distinct spatiotemporal expression dynamics during fruit development: *MaLAR* expression progressively increased throughout fruit maturation, whereas *MaANR* expression reached its maximum at the color-turning stage. *In vitro* enzymatic assays unequivocally confirmed that *MaLAR* and *MaANR* catalyze the formation of catechin and epicatechin, respectively. *In vivo* functional validation further substantiated their roles, as VIGS-mediated silencing of either gene significantly reduced total PA content in mulberry leaves, while heterologous overexpression in *Arabidopsis* markedly enhanced PA accumulation in the seed coat.

In conclusion, the converging evidence from *in vitro* enzymatic characterization, *in vivo* gene silencing, and heterologous overexpression firmly establishes *MaLAR* and *MaANR* as essential positive regulators of PA biosynthesis in mulberry. These findings not only deepen our understanding of the molecular mechanisms governing secondary metabolism in mulberry but also provide valuable molecular targets and theoretical guidance for modulating PA content and improving fruit quality through molecular breeding and metabolic engineering.

## Data Availability

The datasets presented in this study can be found in online repositories. The names of the repository/repositories and accession number(s) can be found in the article/**Supplementary Material**.
