# Supplementary material for: Characterization of two reductases MaLAR and MaANR revealed their roles in proanthocyanidin biosynthesis in mulberry
Source: Front Plant Sci. 2026 Jan 21;16:1760417. doi: 10.3389/fpls.2025.1760417 (PMC12868198; doi:10.3389/fpls.2025.1760417)
Supplement: Supplementary file 1 [file Table1.docx]

**Supplementary**

**Supplementary Fig. S1 PCR amplification of *MaLAR* and *MaANR* genes from mulberry.**
(A) Agarose gel electrophoresis of the PCR-amplified *MaLAR* gene. Lane M: DL2000 DNA marker; Lane 1: *MaLAR* (969 bp). (B) Agarose gel electrophoresis of the PCR-amplified *MaANR* gene. Lane M: DL2000 DNA marker; Lane 1: *MaANR* (1014 bp).


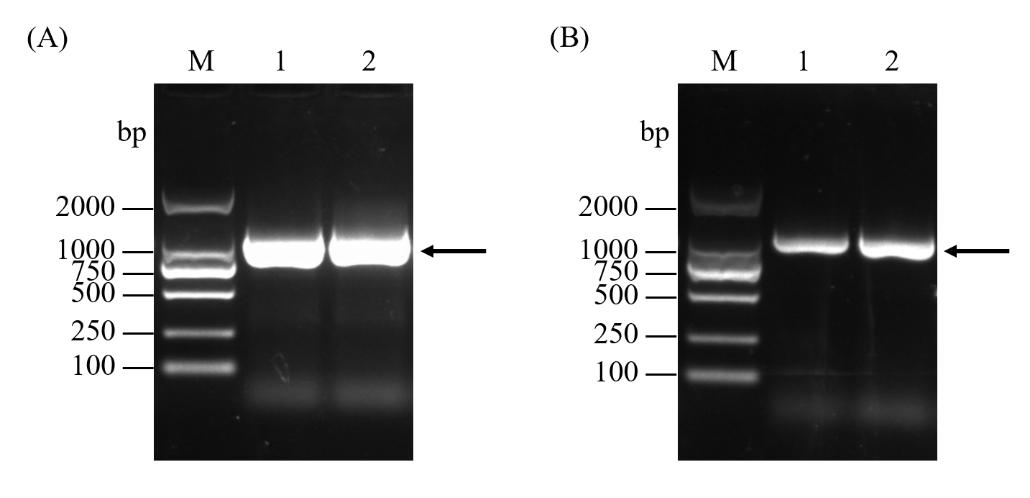


*MaANR*

M

2000bp

1000bp

750bp


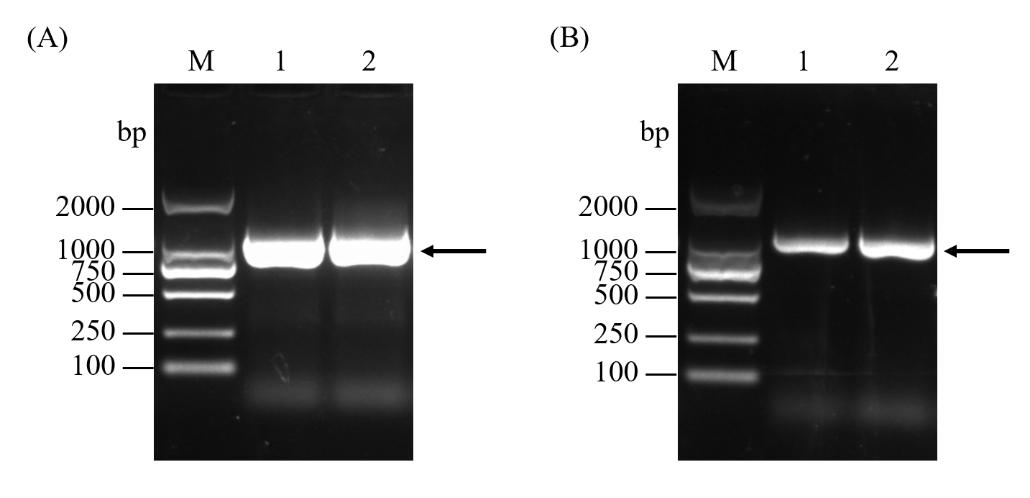


M

*MaLAR*

2000bp

1000bp

750bp

**(A)**

**(B)**

**Supplementary Fig. S2 SDS–PAGE analysis of recombinant pGEX-4T-1–*MaLAR* and pET-28a–*MaANR* proteins.**

(A) pGEX-4T-1–*MaLAR*; (B) pET-28a–*MaANR*; M: protein molecular-weight marker;1: uninduced sample (without IPTG);2: IPTG-induced sample;3: supernatant after ultrasonic disruption;4: flow-through fraction after low-temperature incubation;5: pre-wash fraction;6: post-wash fraction;7–9: elution fractions 1, 2, and 3.


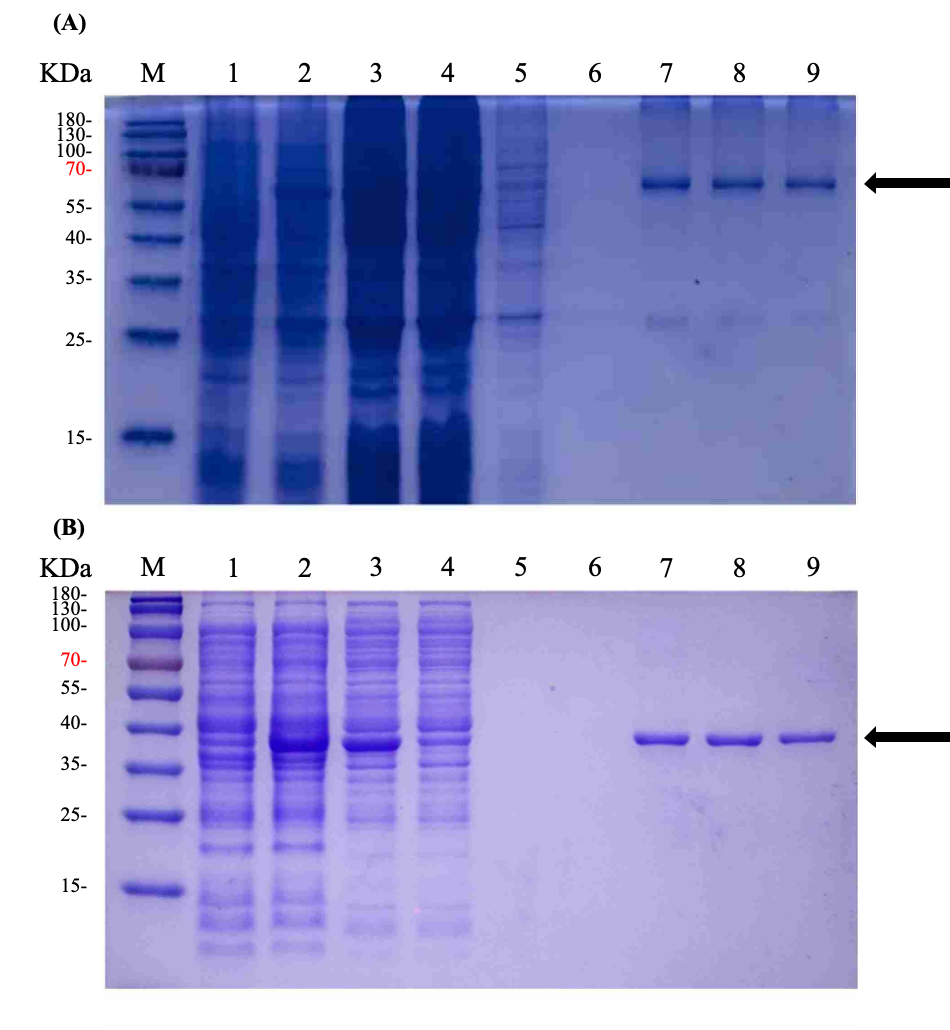


**Supplementary Fig. S3** **Phenotypes of mulberry seedlings under UV illumination following VIGS treatment.**
(A) Control seedlings without VIGS treatment. (B) *MaANR*-silenced seedlings (*MaANR*-VIGS). (C) *MaLAR*-silenced seedlings (*MaLAR*-VIGS).


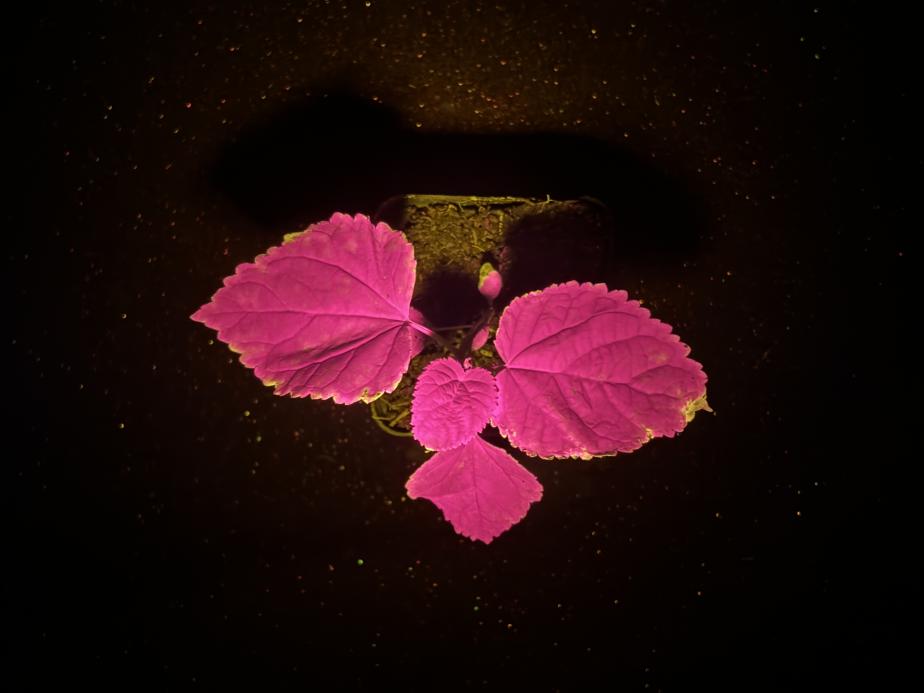

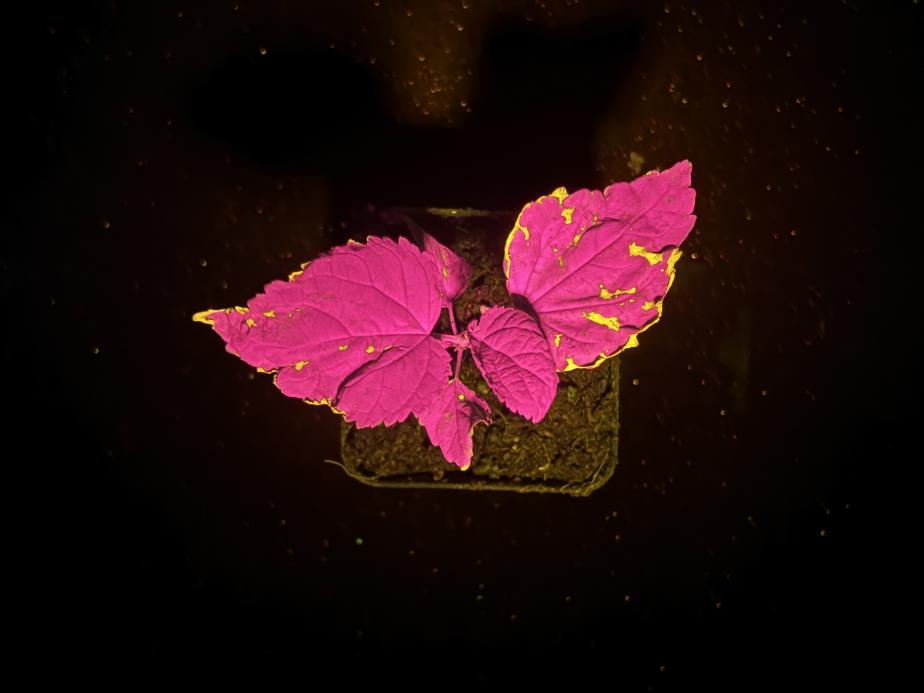

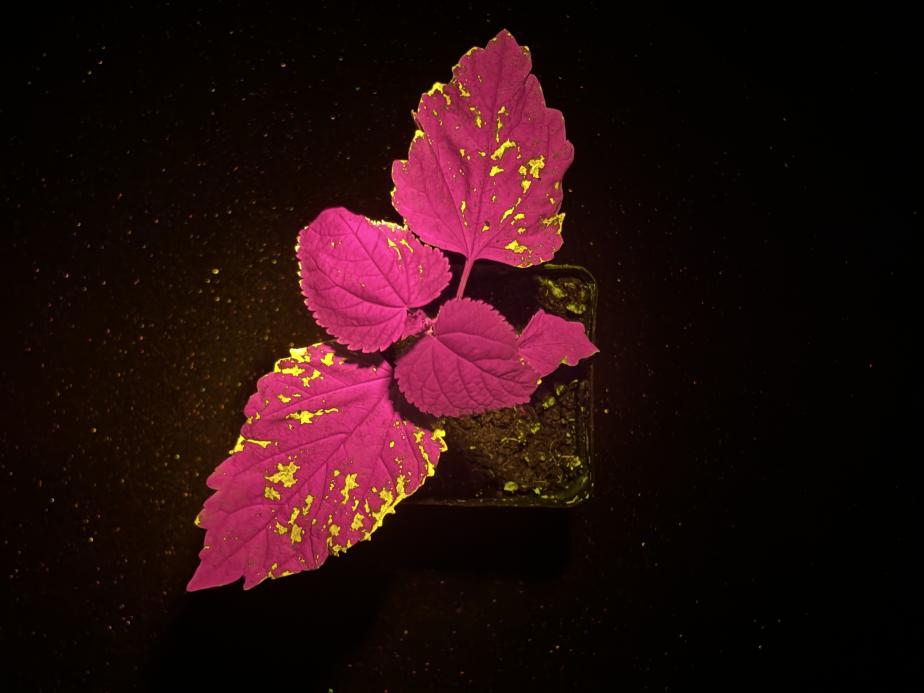


**(A)**

**(B)**

**(C)**

**CK**

***MaANR*-VIGS**

***MaLAR*-VIGS**

**Supplementary Table. S4 Sequences of primers used in this study**

| **Primers sequence for cloning *MaLAR* and *MaANR* genes in mulberry** | |
| --- | --- |
| MaLAR-F | ATGAGTGGCTTAGCTTCAACT |
| MaLAR-R | CTAGTTGTGAGAAGGAGCTCTC |
| MaANR-F | ATGGCCACTCAGACCATCGT |
| MaANR-R | TCAGATGTGAAGCAATCCTTTAGTC |
| **qRT-PCR primers sequence for cloning *MaLAR* and *MaANR* genes in mulberry** | |
| Actin-F  Actin-R | CATTGTAGGTCGTCCCCGTC  TTCTTCAGGGGCAACACGAA |
| MaLAR-QF | GGTTGCATTGAAGACCAACCATT |
| MaLAR-QR | AGGGCCAACCAGCAATAGAGT |
| MaANR-QF | AACTGAGGAAGGCAGCTTCG |
| MaANR-QR | GATTGAAACAGCTGCAGCAG |
| **VIGS primers sequence for cloning *MaLAR* and *MaANR* genes in mulberry** | |
| VIGS-MaLAR-F  VIGS-MaLAR-R | CAGCATCCCCACCTATATTCT  GATAGTGCCGGCTCCACT |
| VIGS-MaANR-F  VIGS-MaANR-R | GGGCTACCCTGTGTCCAA  CAGCAAATGTAACGACCAGAAG |
| NC-VIGS-MaLAR-F | AGTGGTCTCTGTCCAGTCCTCAGCATCCCCACCTATATTCT |
| NC-VIGS-MaLAR-R | GGTCTCAGCAGACCACAAGTGATAGTGCCGGCTCCACT |
| NC-VIGS-MaANR-F  NC-VIGS-MaANR-R | AGTGGTCTCTGTCCAGTCCTGGGCTACCCTGTGTCCAA  GGTCTCAGCAGACCACAAGTCAGCAAATGTAACGACCAGAAG |
| **Primers used to verify *MaLAR* and *MaANR* overexpression in *Arabidopsis*** | |
| Atactin7-QF | ACATCGTTCTCAGTGGTGGT |
| Atactin7-QR | GCTGAGGGATGCAAGGATTG |
| MaLAR-QF | GGTTGCATTGAAGACCAACCATT |
| MaLAR-QR | AGGGCCAACCAGCAATAGAGT |
| MaANR-QF | AACTGAGGAAGGCAGCTTCG |
| MaANR-QR | GATTGAAACAGCTGCAGCAG |
| **Primer sequences of prokaryotic expression vectors for the *MaLAR* and *MaANR*** | |
| pGEX-MaLAR-F | CGCGTGGATCCCCGGAATTCATGAGTGGCTTAGCTTCAAC |
| pGEX-MaLAR-R | GCCGCTCGAGTCGACCCGGCTAGTTGTGAGAAGGAGCTCTC |
| pET-28a-MaANR-F | CAGCAAATGGGTCGCGGAATGGCCACTCAGACCATCGT |
| pET-28a-MaANR-R | GACGGAGCTCGAATTCGGATCAGATGTGAAGCAATCCTTTAGTC |
| **Primers sequence of *MaLAR* and *MaANR* genes with NC junction in mulberry** | |
| NC-MaLAR-F | AGTGGTCTCTGTCCAGTCCTATGAGTGGCTTAGCTTCAACT |
| NC-MaLAR-R | GGTCTCAGCAGACCACAAGTCTAGTTGTGAGAAGGAGCTCTC |
| NC-MaANR-F | AGTGGTCTCTGTCCAGTCCTATGGCCACTCAGACCATCGT |
| NC-MaANR-R | GGTCTCAGCAGACCACAAGTTCAGATGTGAAGCAATCCTTTAGTC |
